# An Information Theory Inspired Real-Time Self-Adaptive Scheduling for Production-Logistics Resources: Framework, Principle, and Implementation

**DOI:** 10.3390/s20247007

**Published:** 2020-12-08

**Authors:** Wenchao Yang, Wenfeng Li, Yulian Cao, Yun Luo, Lijun He

**Affiliations:** 1 School of Logistics Engineering, Wuhan University of Technology, Wuhan 430070, China; 1030990690@whut.edu.cn (W.Y.); yunluo@whut.edu.cn (Y.L.); helj@whut.edu.cn (L.H.); 2 School of Aviation, University of New South Wales, Sydney, NSW 2052, Australia; yulian.cao@unsw.edu.au

**Keywords:** smart job-shop, production-logistics resources, information theory, self-adaptive scheduling

## Abstract

The development of industrial-enabling technology, such as the industrial Internet of Things and physical network system, makes it possible to use real-time information in production-logistics scheduling. Real-time information in an intelligent factory is random, such as the arrival of customers’ jobs, and fuzzy, such as the processing time of Production-Logistics Resources. Besides, the coordination of production and logistic resources in a flexible workshop is also a hot issue. The availability of this information will enhance the quality of making scheduling decisions. However, when and how to use this information to realize the adaptive collaboration of Production-Logistics Resources are vital issues. Therefore, this paper studies the above problems by establishing a real-time reaction scheduling framework of Production-Logistics Resources dynamic cooperation. Firstly, a real-time task triggering strategy to maximize information utilization is proposed to explore when to use real-time information. Secondly, a collaborative method for Production-Logistics Resources is studied to explore how to use real-time information. Thirdly, a real-time self-adaptive scheduling algorithm based on information entropy is utilized to obtain a stable and feasible solution. Finally, the effectiveness and advancement of the proposed method are verified by a practical case.

## 1. Introduction

With the increasing demand for product customization, enterprise orders are also developing towards diversification and small-batch [1]. The direct involvement of customers in the whole product life cycle significantly increases the uncertainty of production processes. Meanwhile, changes in customer demand not only affect the addition/deletion of orders, but the resulting changes in the production process also put forward higher requirements for the dynamic organization ability of Production-Logistics Resources in the job-shop. In this case, the improvement of product personalization dramatically increases the difficulty of production scheduling. Small batches and random order arrival time increase the complexity of scheduling problems. Production and logistics equipment, due to lack of dynamic collaboration, will lead to an increase in waiting time and a lot of waste of energy. The real-time changes of production tasks in a smart job-shop propose higher requirements for the dynamic response capability of Production-Logistics Resources.

The rapid progress of Industry 4.0 has led to the following new features in some modern industrial production organizations [2]. (1) The communication capacity and automation level of Production-Logistics Resources are getting higher and higher. Especially, mobile devices such as Automated Guided Vehicles (AGVs) with the networking capacity and computing capacity becoming new production scheduling factors. (2) The development and application of technologies such as the Internet of Things (IoT) and Cyber-Physical Systems (CPS) have made the manufacturing resources in smart factories (e.g., machines, AGVs, intelligent forklifts, etc.) ever more intelligent [3]. On the one hand, the application of intelligent resources in the factory makes manufacturing intelligent possible. On the other hand, intelligent resources will also bring fuzzy operation time, including transportation time caused by the uncertainty of the impact of AGVs obstacle avoidance and human factors on the production operation time during human-machine cooperation [4]. (3) The flexibility of production organization is the basis for enterprises to meet personalized customer needs. This not only requires the factory’s production factors to have the ability to dynamically organize to meet production demands, but also raises the dynamic adaptability requirements of production organization for security, flexibility, and agility [5].

Although the application of enabling technology in manufacturing has made significant progress, the following problems still exist in the job-shop scheduling in real-time dynamic environment. (1) Simply introducing intelligent resources into intelligent factories without efficient management methods can not reflect the advantages of intelligent manufacturing [6]. This is because it may cause conflicts and confrontations among intelligent resources due to their parallelism and sudden behavior. How to rationally organize logistics resources and production resources to perform real-time tasks in the job-shop is based on the realizing of intelligent production [7]. (2) With the increasing application of enabling technology in factories, real-time reaction scheduling has become a hot issue for scholars [8]. However, most scholars pay attention to the random arrival of orders and ignore the impact of fuzzy working hours on job-shop. (3) In order to realize the real-time reaction scheduling of Production-Logistics Resources in intelligent job shop, it is necessary to study when the Production-Logistics Resources in the system provide services. Moreover, how to provide services according to the current task requirements and their service capabilities is a critical issue to be addressed in a discrete manufacturing system.

In light of the above-described problems, this paper proposes a real-time reaction scheduling framework for production-logistics resources. The real-time reaction scheduling framework includes three parts: (1) The capacity model of production logistics resources is constructed. The multi-attribute of workshop resource is the basis of multi-resource adaptive scheduling. (2) A real-time task triggering mechanism for maximizing information efficiency is proposed. This solves the problem of when to allocate real-time tasks in different degrees of disturbance. (3) A production logistics resources real-time adaptive scheduling method is proposed. This method achieves the result of global optimization through adaptive task allocation.

The remainder of this paper is organized as follows. Section 1 reviews the relevant studies. Section 2 demonstrates the architecture of the proposed method. The Real-time Self-adaption Scheduling (RSS) method is presented in Section 3. In Section 4, the validity of the proposed method is verified. Finally, conclusions are drawn in Section 5.

## 2. Related Work

Related work includes the following parts: The dynamic scheduling of random orders, job-shop scheduling with fuzzy operation time, real-time reaction scheduling, and multi-resource cooperative scheduling.

Flexible Job-shop Scheduling Problem (FJSP) is a fundamental problem in the manufacturing industry. In the past decade, the industry and academia have carried out much research on FJSP and developed various response methods for disturbance factors [9]. In the intelligent factory, the random arrival of the workpiece is one of the essential disturbance factors. The researchers in [10] defined the job arrival rate λ as Poisson random variables. By analyzing the performance of different work arrival rate λ, the proposed method’s superiority is tested. After that, scholars study how to reschedule new jobs with random arrival based on the performance and stability of scheduling [11]. In reference [12], a hybrid genetic algorithm method is proposed to minimize the generation time. Minimizing total energy cost and total delay in a workshop with random arrival job is studied in [13].

Fuzzy Job Shop Scheduling Problem (FJSSP) [14] is an extension of job shop scheduling by fuzzy processing time or fuzzy precedence constraints, etc. The processing time of equipment in a factory is generally assumed to be a definite value. This assumption is idealistic and does not conform to the actual situation because there are uncertain disturbance factors in the factory of the real world. In [15], the uncertain processing time was modeled by Triangular Fuzzy Number (TFN) function, and an effective hybrid cooperative coevolution algorithm for the minimization of fuzzy makespan is proposed. Due to the operation rule of TFN being a necessary condition to establish a scheduling table, TFN is usually used to indicate processing conditions [14]. In [16], a novel algorithm called hybrid multi-verse optimization is proposed to address the fuzzy flexible job-shop scheduling problem.

The dynamic scheduling method can respond to low-frequency abnormal events, but it is challenging to meet the production scenarios with a high disturbance [8]. Industry 4.0, with its powerful real-time data acquisition capability, can bridge the gap between the actual manufacturing situation and the mathematical model [17]. In [18], the time point of the new scheduling rule is dynamically selected according to the real-time information, and real-time reaction scheduling is realized. Under the concept of manufacturing as a service, the theory and method of multi-attribute decision-making are introduced to study service innovation-oriented to product life cycle based on real-time information [19]. In [20], a collaborative service method of manufacturing resources for future intelligent manufacturing scenarios based on the hyper network’s scheduling model is proposed. In [21], the researchers believe that the traditional task allocation method of manufacturing workshops can not meet the production management requirements of the intelligent job-shop, so it is necessary to construct an adaptive task allocation method.

Stuart and Kozan [8] formulated the multiple operating rooms surgical sequencing problem with surgeons and operating rooms are constrained resources as a resource-constrained parallel-machine scheduling problem. A real-time reactive scheduling strategy is proposed to realize adaptive scheduling. In [22], the problem of multi-resource collaboration is studied. The author defines primary resources (such as machines) and secondary auxiliary resources (such as personnel). The optimal scheduling scheme is generated by constructing a mixed-integer programming model. The literature [23] points out that logistic activities in manufacturing occupy more than 90% of the production time and production logistic collaboration are significant to realize intelligent manufacturing. In [24], an adaptive collaborative method of a production logistics system based on Petri net is proposed. Zhang et al. [25] proposed a production logistics collaborative method based on target cascade to realize self-organization configuration.

From the above literature, we noticed the following points: (1) The above researchers studied the unexpected arrival and fuzzy processing time of jobs. Most of them pay attention to the scheduling of production resources constrained [26] and lack attention to the smart workshop’s logistics resources constrained. AGV is widely used in intelligent workshop, which makes logistics resources more critical. It is necessary to do more research on production flow collaborative scheduling in a high disturbance environment. (2) Especially in the field of real-time reaction scheduling, a few scholars have begun to explore collaborative scheduling methods of production logistics. However, real-time reaction scheduling with fuzzy operation time is rarely studied.

Based on the above analysis, we provide an adaptive scheduling method of production-logistics resources cooperation to reduce energy consumption, shorten the makespan, and improve customer satisfaction. Firstly, this real-time reactive scheduling method needs to analyze the historical data to determine the appropriate model parameters and assumptions. Based on the case factory’s historical data, we assume that the task arrival obeys Poisson distribution, and the operation time of the workshop resource obeys the triangular distribution. Secondly, this real-time reactive scheduling method needs the support of real-time data to realize the real-time task allocation of an intelligent job shop. Here, we assume the real-time status of real-time tasks and workshop resources issued by the industrial cloud platform as input. Through the real-time reactive scheduling framework proposed in this paper, we realize the real-time reactive scheduling of high dynamic workshops and adjust the task allocation’s evaluation function adaptively according to the task requirements to maintain the feasibility.

## 3. Framework of Real-Time Self-Adaptive Scheduling

The development of enabling technology in industry 4.0 makes manufacturing more intelligent. In particular, the production logistics association is an inevitable mode of future production. Random orders and fuzzy operation time are also inevitable problems in the workshop. In this paper, the real-time reaction scheduling method is used to solve the three problems (production logistics coordination, random order, and fuzzy operation time). This section shows the characteristic description of the problem and the architecture of the proposed method.

### 3.1. Characteristics and Description of the Problem

To facilitate reading and understanding, some commonly-used math notations in this article are listed in Table 1.

The core of real-time reaction scheduling problem for the intelligent job-shop with multiple disturbances is a matching problem between manufacturing resources, including production resources and logistics resources, according to their status and production tasks with specific process requirements. In the environment of the Industrial Internet of Things (IIoT), (1) although the states of Production-Logistics Resources can be perceived and monitored in real-time, the operation time of Production-Logistics Resources have fuzzy characteristics. (2) Jobs (i.e., customized orders) arrive in real-time and randomly.

Different from the traditional scheduling problem, the research objective of this paper is a multi-resource real-time reaction scheduling problem in a smart job-shop. The manufacturing problem to be solved in this study has the following significant characteristics.

- *Scheduling objects.* Most of the existing scheduling methods mainly focus on production resources, while the scheduling of workshop logistics resources is simplified. For example, the research on production usually only considers the scheduling of machines and regards logistics processing as a boundary condition [19]. The logistics processing systems pay more attention to issues such as route optimization [27]. However, in the environment of Industry 4.0, more and more intelligent manufacturing equipment such as smart machines and smart vehicles will be used in the job-shop. Scheduling resources include not only production equipment, but also logistics equipment, such as AGVs. The Production-Logistics Resources in the real-time production process influence and restrict each other. The construction of Production-Logistics Resources dynamic cooperation mode is the basis of Production-Logistics Resources adaptive collaboration;
- *Execution.* Due to the existence of fuzzy factors of the operation time, the essence of each task assignment is based on the prediction of the service capacity of the current Production-Logistics Resources. Therefore, the results of each task execution cannot be accurately predicted.

The real-time reaction scheduling problem can be stated as follows. Given a set of jobs $job_{set}\;=\;\left\{job^{1},job^{2},\cdot \cdot \cdot ,job^{N}\right\}$, a set of machines $M\;=\;\left\{m_{1},m_{2},\cdot \cdot \cdot ,m_{J}\right\}$, and a set of AGVs $V\;=\;\left\{v_{1},v_{2},\cdot \cdot \cdot ,v_{I}\right\}$. Based on the existing job shop scheduling problems [9,28], in this study, assumptions are used as follows:

- As time goes on, random jobs arrive in the job-shop. Moreover, the distribution of job arrival process follows the Poisson distribution;
- The operation time of Production-Logistics Resources obeys the fuzzy triangular distribution $\left[tf_{1},tf_{2},tf_{3}\right]$, where $tf_{2}$ is the estimated operation time of Production-Logistics Resources. $tf_{1}$ and $tf_{3}$ denote the upper and lower bounds of the fuzzy intervals;
- Since the communication cost is not considered, the waiting energy consumption of AGVs is zero;
- The loading and unloading time of AGVs is regarded as logistics time;
- Machines and AGVs only process one Work In Progress (WIP) at a time.

### 3.2. The Framework of Real-Time Self-Adaption Scheduling

**Figure 1:**
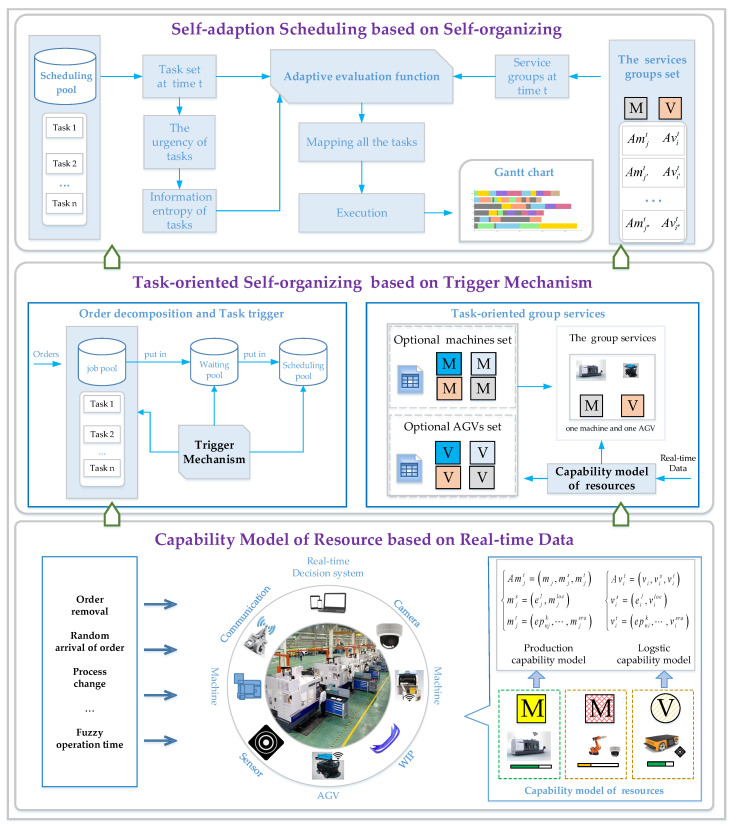
The framework of real-time reaction scheduling for Production-Logistics Resources.

The framework of real-time reaction scheduling based on Multi-resources Dynamic Collaboration (MDC) in the job-shop is shown in Figure 1. It includes three parts: (1) The capability model of smart Production-Logistics Resources based on real-time data to realize the real-time perception of resources. (2) Real-time Task-oriented Self-organizing (RTS) of job-shop resources based on the Trigger Mechanism (TM) to maximize the utilization of information in the intelligent job-shop. (3) RSS based on RTS to realize adaptive scheduling. The functions of each module are described below.

In the environment of Industry 4.0, Production-Logistics Resources are becoming ever more intelligent in the process of realizing smart manufacturing. By embedding RFID, sensors, processors, and other intelligent modules into the equipment of job-shop, the intelligent communication and execution ability of Production-Logistics Resources in a smart job-shop are realized.

In a smart job-shop, random arrival jobs and real-time dynamic characteristics of a resource status put forward higher requirements for the flexibility of the real-time collaboration of Production-Logistics Resources. Therefore, in order to deal with the uncertainty of task arrival and fuzzy operation time in job-shop, a dynamic self-organization strategy of Production-Logistics Resources is proposed to reflect real-time tasks. This strategy is oriented to real-time task requirements in the job-shop, which combines and matches the optional service resources according to the real-time service capability of Production- Logistics Resources to achieve the task-oriented real-time self-organization of Production-Logistics Resources. The key to realizing RTS is when and how to release tasks, which is called a task trigger mechanism. As shown in Figure 1, when entering the job-shop, the job will be divided into the smallest atoms (tasks), and each task requirement contains a set of services, namely logistics services and processing services [29]. Therefore, the trigger mechanism proposed in this paper is based on the smallest production-logistics collaboration granularity as the triggering measurement.

The synchronization of production-logistics is the key to the intelligence of job-shop [25]. Unlike the traditional scheduling strategy, the real-time reaction scheduling strategy proposed in this paper is no longer to schedule production tasks or smart logistics separately, but to consider the combination of current production and logistics to optimize the service model (i.e., RTS) composed of production-logistics services. Therefore, the proposed RSS can not only deal with the real-time changes of production tasks, but also continuously correct the deviation between scheduling and execution.

## 4. Real-Time Reaction Scheduling Strategy

This section displays the key elements of RSS, i.e., the resource encapsulation method, trigger mechanism based on real-time data, and scheduling method.

### 4.1. Capability Model of Production-Logistics Resources

The construction of the capability model of manufacturing resources in the IIoT environment includes two parts, namely the properties of the resources and their real-time status. The former includes the scope and characteristics of business capability, energy consumption, and service quality. The latter includes production and logistics, anomaly detection, dynamic queues, service load, and status in the service process. The real-time perception of key manufacturing resource status in a smart job-shop is based on the building establishment of a smart resource model. The manufacturing cost of WIP includes raw material cost and maintenance cost, which are inherent cost and will not change due to production plans. Time cost includes processing time and handling time. The processing time depends on the service capacity of the equipment arranged for the process production, which may change due to different production scheduling. The handling time not only depends on the service capacity of the arranged equipment, but also depends on the processing positions of the adjacent operations of WIP, which may change due to different production schedules. It is assumed that AGVs have the same speed and energy consumption in this study, so the difference of their positions is the only factor that causes the difference in picking/delivery time. In order to better manage the real-time status data of the critical resources, the capability model of processing equipment and handling equipment is built, as shown below.

The capability model of production resources has three characteristics, namely equipment number, static attribute, and real-time status attribute.

(1) $$\begin{array}{c} Am_{j}^{t}=\left(m_{j},m_{j}^{s},m_{j}^{t}\right) \end{array}$$

The static attributes include idle power $e_{j}^{I}$ and locality information $M_{j}^{loc}$ of machine resources. The real-time attributes include the type of service $S_{j}^{t}$ that the machine can provide, power $ep_{nj}^{k}$, service time $p_{nj}^{k}$, service queue $m_{j}^{que}$, and $m_{j}^{sta}$ resource status of machine resources. The capability model of logistics resources also has the equipment number, static attribute, and real-time status attribute characteristics.

(2) $$\begin{array}{c} Av_{i}^{t}=\left(v_{i},v_{i}^{s},v_{i}^{t}\right) \end{array}$$

The static attributes include idle power $e_{i}^{I}$ and speed $\dot{v_{i}}$. The real-time attributes include the type of service $S_{i}^{t}$, power $ep_{ni}^{k}$, service time $p_{ni}^{k}$, service queue $v_{i}^{que}$, locality information of logistics resources $v_{i}^{loc}$, and logistics resources status $v_{i}^{sta}$.

### 4.2. Scheduling Trigger Mechanism Based on Real-Time Tasks

The most significant difference between real-time reaction scheduling and traditional scheduling is real-time task allocation. When only production schedule is considered, due to the high efficiency and high speed of real-time reaction scheduling algorithms, the time point for real-time allocation can be at the end of the previous process [30]. At this point, assigning real-time tasks has obvious advantages. For example, the end time of the current task and the status of the current optional service equipment can be accurately known, so that the fuzziness of time nodes caused by the status estimation can be remarkably avoided. However, the result of production scheduling will directly affect logistics services when logistics status in the job-shop is considered. The status of logistics services will also affect the orderly production and processing. Therefore, when considering the interaction and influence of production and logistics in the smart job-shop, the triggering time of real-time reaction scheduling is the critical factor affecting scheduling performance.

There is no doubt that the scheduling problem of Production-Logistics Resources is NP-hard in the smart job-shop. Furthermore, the random arrival of orders and the fuzziness of operation time make this problem more complicated. In order to realize the dynamic collaboration of Production-Logistics Resources in a multi-disturbance environment, this paper uses information theory to study the mechanism of this complex problem. Based on the existing research on information theory [31] and the characteristics of intelligent job-shop.

**Definition** **1**(Information Utility)**.** *The information utility is related to the time when the information is generated and it gets increasingly smaller as time goes by.*

The real-time information of smart job-shop is the carrier of the job-shop status description. When the real-time information of smart job-shop is collected, this is the most valuable moment of the information. As time goes by, the information utility will gradually decrease or even disappear. Taking pre-scheduling as an example, the global scheduling is performed at the beginning. It is assumed that the information utility gained at the beginning will not change over time. In other words, there will be no disturbance during the manufacturing process and all resources will operate in strict accordance with the expected plan. Then, in terms of the effect of production optimization, the pre-scheduling must be at the forefront of global optimization solutions. However, due to uncertain factors in the production process, such as the fuzziness of production time and handling time, equipment failures, and irregular order additions or deletions, excellent scheduling results will become unsatisfactory to a large extent, or even infeasible.

**Definition** **2**(Maximum information utility)**.** *The maximum information utility is to maximize the utility of information to promote the optimization of the production process by making use of the current information at a reasonable time.*

On the one hand, in the real-time reaction scheduling of smart job-shop with production-logistics collaboration, the handling equipment responsible for logistics tasks pre-computes the time when it arrives at the delivery point, and predicts the completion time of the current task, so that the actual delivery time can be estimated. On the other hand, the handling equipment in charge of logistic tasks needs to know the ending point (the location of the service machine) of the task, so it is necessary to predict the available time of the terminal processing equipment.

**Figure 2:**
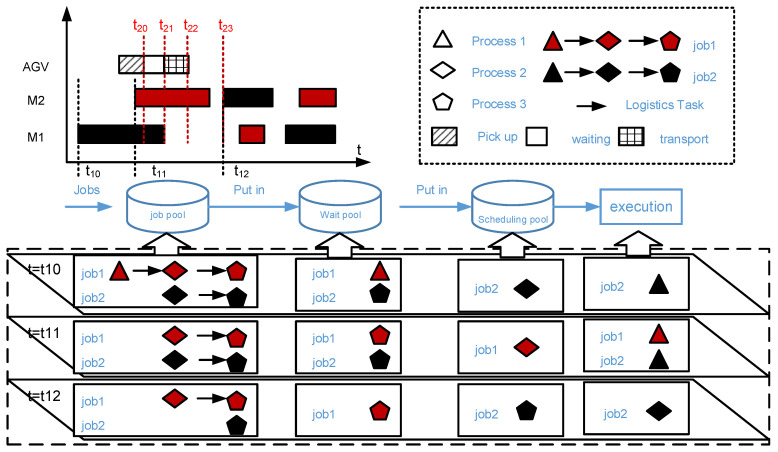
The trigger mechanism of real-time reaction scheduling based on real-time data.

Figure 2 uses two jobs, two machines, and one AGV as an example to describe the task status, resource status, and status transition logic at different time nodes. Among them, each workpiece contains three processes. Each process can be processed on different machines. And the processing time is different. There are four states for tasks: Task pool, waiting pool, and scheduling pool and executed. In this paper, all jobs will be triggered immediately after they reach the workshop from the cloud. In other words, the first process of each job will be put into the scheduling pool when the job arrives. At this time, the second process of the job will be put into the waiting pool and the remaining processes of the job will be put into the task pool. When a task in the waiting pool is triggered, it will be placed in the scheduling pool. Then, it will use the adaptive task allocation method proposed in this article to find the most suitable Production-Logistics Resources for this task. Finally, it will join the service queue of Production-Logistics Resources and wait to be executed. It is worth emphasizing that this article considers the coordinated execution of Production-Logistics Resources, so a handling task accompanied each processing task. A task trigger strategy that only considers production resources can trigger the next operation when it is completed [30]. However, in the production logistics collaboration scenario, task triggering must be performed before the end of the production task because the pickup time of logistics resources must be considered. Therefore, selecting the task trigger time according to the system-like disturbance state is a core scientific problem to be solved in this paper.

The proposed trigger mechanism is that the scheduling of the current process will be triggered at the processing point of its previous process. On the one hand, triggering the next process in advance will help avoid the waiting time of the WIP when AGV takes conduct delivery task. On the other hand, using the processing event with the smallest granularity (adjacent process) as the trigger event can maximize the effective use of information and avoid the failure of feasible solutions due to premature triggering of subsequent process scheduling. In order to explore the impact of trigger time on real-time reaction scheduling results under the high disturbance environment, as shown in Figure 2, the proposed trigger mechanism contains four strategies.

For ease of understanding, the following is a simple trigger at the Machine Start Processing strategy (MSP). Both $job_{1}$ and $job_{2}$ have three processes, including $O_{1}^{1}$, $O_{1}^{2}$, $O_{1}^{3}$, and $O_{2}^{1}$, $O_{2}^{2}$, $O_{2}^{3}$. At the time $t_{10}$, $O_{2}^{1}$ starts to be processed on the machine $m_{1}$. At this time, the processing task $O_{2}^{2}$ is triggered and moved from the waiting pool to the scheduling pool. $O_{2}^{3}$ is moved from the job pool to the waiting pool. Task $O_{2}^{2}$ is released to the set of optional services $m_{j}^{t}$ at the same time. A real-time reaction scheduling algorithm evaluates the collaborative service capacities of production equipment and logistics equipment, and selects the best services combination to complete the task $O_{2}^{2}$. At the time $t_{11}$, $O_{2}^{1}$ continues to be processed on the machine $m_{1}$, and $O_{1}^{1}$ starts to be processed on the machine $m_{2}$. At this time, $O_{1}^{2}$ is triggered and moved from the waiting pool to the scheduling pool. $O_{1}^{3}$ is moved from the job pool to the waiting pool. In the same way, the scheduling algorithm is triggered to match services combination. At the time $t_{12}$, $O_{1}^{1}$ and $O_{2}^{1}$ have been completed and $O_{2}^{2}$ starts to be processed on the machine $m_{2}$. At this time, $O_{2}^{3}$ is triggered. It is moved from the waiting pool to the scheduling pool to match services combination by algorithms.

### 4.3. Real-Time Reaction Scheduling Algorithm

Different from the traditional scheduling algorithm, the production-logistics collaborative real-time reaction scheduling algorithm in smart job-shop not only considers the matching between the production task and production equipment, but also considers the matching between logistics service and demand. Smart production equipment and smart logistics equipment are introduced into a smart job-shop. On the one hand, the intellectualization of Production-Logistics Resources increases the visualization and controllability in the production process and provides the possibility for the intellectualization of manufacturing. On the other hand, these intelligent Production-Logistics Resources increase the difficulty of manufacturing system management and the complexity of the scheduling problem. In a multi-disturbance production scenario, managing production resources dynamically is the basis of realizing smart manufacturing.

#### 4.3.1. Objective Functions of Smart Job-Shop

The objective of the smart job-shop level is to minimize the average task delay, the job completion time, and the total energy consumption of Production-Logistics Resources.

Minimize the makespan $\left(C_{max}\right)$:

(3) $$\begin{array}{c} Min\;C_{max}=max\left\{C^{n}\right\} \end{array}$$

Minimize the total energy consumption $\left(E\right)$:

(4) $$\begin{array}{c} Min\;E=\sum_{i=1}^{I}E_{v}^{i}+\sum_{j=1}^{J}E_{m}^{j} \end{array}$$

Minimize the mean tardiness $\left(MT\right)$:

(5) $$\begin{array}{c} Min\;MT=\frac{1}{N}\times \sum_{n=1}^{N}L^{n} \end{array}$$

where $E_{v}^{i}$ denotes total energy consumption of AGV $v_{i}$, $E_{m}^{j}$ denotes total energy consumption of the machine $m_{j}$, and $L_{n}$ denotes the lateness of $job^{n}$.

#### 4.3.2. Objective Functions of Real-Time Self-Adaption Collaboration

In the multi-objective real-time reaction scheduling method, the weight method is usually used as the evaluation standard of task allocation. The weight of the evaluation index for traditional scheduling rules in real-time reaction scheduling needs to be adjusted manually [25]. This method is not suitable for intelligent job shops with multiple interferences. Due to random arrive jobs and fuzzy operation time, it is impossible to predict weight values through simulation. Since each simulation is a unique case, nothing else can duplicate. To avoid the shortcomings of the traditional rule-based real-time reaction scheduling method, an adaptive scheduling approach is proposed in this paper. The real-time self-adaption scheduling algorithm has been verified in our previous studies, which combine with an actual case [29]. The interferences include random orders arrivals and fuzzy operation time, which makes the manufacturing scenario more complex and changeable. In order to adapt to a more elaborate production environment, an improved self-adaption algorithm is put forward as follows:

(1) In order to strengthen the dynamic collaboration among resources, such as production resources and logistic resources, participate in the task allocation and execution in the form of groups:
  (6) $$\begin{array}{c} G_{u}^{t}=\left\{\left(v_{i},m_{j}\right)\right\},v_{i}\in \bar{V},m_{j}\in \bar{M} \end{array}$$
(2) The service capacity of each group $g_{y}$ includes service time $T^{y}$ and energy consumption $W^{y}$ for task $O_{n}^{k}$:
  (7) $$\begin{array}{c} T^{y}=T_{pic}^{y}+T_{sen}^{y}+T_{pre}^{y}+T_{c}^{y} \end{array}$$
  (8) $$\begin{array}{c} W^{y}=W_{pic}^{y}+W_{sen}^{y}+W_{pre}^{y}+W_{c}^{y} \end{array}$$
  where, $T_{pic}^{y}$ denotes time cost of picking up WIP. $T_{sen}^{y}$ denotes time cost of delivering the WIP to the appropriate location. $T_{pre}^{y}$ denotes the processing time. $T_{c}^{y}$ denotes time cost of Production- Logistics Resources collaboration, such as the waiting time of pick-up and processing. $W_{pic}^{y}$ denotes energy consumption of picking up WIP. $W_{sen}^{y}$ denotes energy consumption of delivering the WIP to the appropriate location. $W_{pre}^{y}$ denotes the energy consumption of processing time. $W_{c}^{y}$ denotes the energy consumption of Production-Logistics Resources collaboration, such as the idle power consumption.
(3) In order to estimate the urgency of $O_{n}^{k}$, we use the method of paper [29], which is shown in Equation (9), where $rt\left(O_{n}^{k}\right)$ is the estimation time of remaining and $dt\left(O_{n}^{k}\right)$ is the remaining time of $O_{n}^{k}$. Details can be obtained from our previous work in [29].
  (9) $$\begin{array}{c} U\left(O_{n}^{k}\right)=\frac{rt\left(O_{n}^{k}\right)}{dt\left(O_{n}^{k}\right)} \end{array}$$
  In order to achieve the goal of adaptive scheduling, task tardiness is used to balance operation time and energy consumption index. Equations (10)–(12) are the implementation processes:
  (10) $$\begin{array}{c} NU\left(O_{n}^{k}\right)=\frac{1}{2}+\frac{U\left(O_{n}^{k}\right)-\min\left[U\left(O_{n}^{k}\right)\right]}{2\left\{\max\left[U\left(O_{n}^{k}\right)\right]-\min\left[U\left(O_{n}^{k}\right)\right]\right\}} \end{array}$$
  (11) $$\begin{array}{c} E\left(O_{n}^{k}\right)=-\log_{2}NU\left(O_{n}^{k}\right) \end{array}$$
  (12) $$\begin{array}{c} F=\left[1-E\left(O_{n}^{k}\right)\right]\times \frac{T_{max}^{y}-T^{y}}{T^{y}-T_{min}^{y}}+E\left(O_{n}^{k}\right)\times \frac{W_{max}^{y}-W^{y}}{W^{y}-W_{min}^{y}} \end{array}$$
  In order to avoid the overflow of the definition domain in logarithm operation, Equation (10) guarantees the $NU\left(O_{n}^{k}\right)$ ranges from 0 to 1. Equation (11) denotes adaptive weight based on information entropy of task $O_{n}^{k}$. Equation (12) denotes the adaptive dynamic collaborative evaluation function of Production-Logistics Resources.

#### 4.3.3. The Information Model of the Real-Time System and Mathematics Mechanism Analysis

To facilitate the theoretical analysis, the following three assumptions are adopted:

(1) The tasks in the scheduling pool can be described as $O_{n}^{kb}$ at time *t*, where $O_{n}^{kb}=O_{n}^{k}$, $b\in \left[1,x\right]$;
(2) All tasks in the task pool are attainable, which is $U\left(O_{n}^{k}\right)\leq 1$;
(3) At present, the relevant information value of service matching calculation is only ideal. The system will schedule production according to the assigned tasks and will not change.

According to information theory, the information entropy of the system $H_{sys}^{t}$ is calculated by:

(13) $$\begin{array}{c} H_{sys}^{t}=-\sum_{b=1}^{x}\left[NU\left(O_{n}^{kb}\right)\times \log_{2}NU\left(O_{n}^{kb}\right)\right] \end{array}$$

Based on the information theory of Shannon [32], the amount of information is inversely proportional to the probability of an event. The total information of the scheduling pool is denoted as:

(14) $$\begin{array}{c} I\left(O_{n}^{kb}\right)=-\log_{2}NU\left(O_{n}^{kb}\right)\times rt\left(O_{n}^{kb}\right) \end{array}$$

where, $I\left(O_{n}^{kb}\right)$ is the information amount of $O_{n}^{kb}$. $n_{b}$ is the ratio of the most extensive remaining task to $dt\left(O_{n}^{k}\right)$ in the scheduling pool at time t. Hence, $n_{b}=\frac{\max\left[dt\left(O_{n}^{k}\right)\right]}{dt\left(O_{n}^{k}\right)}\leq 1$. Then, it is easy to get the total amount of information for all tasks in the scheduling pool at time *t*.

(15) $$\begin{array}{c} I_{sch}^{t}=\sum_{b=1}^{x}I\left(O_{n}^{kb}\right)\times n_{b} \end{array}$$

At this time, the information of the scheduling system ($I_{sys}^{t}$) is based on the system entropy and the maximum estimation remaining time. We have:

(16) $$\begin{array}{c} I_{sys}^{t}=H_{sys}^{t}\times \max\left[rt\left(O_{n}^{kb}\right)\right]. \end{array}$$

From the above analysis, we describe the service requirements of tasks and service capabilities of resources in the form of information, namely the information of the system and information of tasks in the task pool. The relationship between task requirements and system service capability can be represented by the ratio of total information of tasks to full information of the scheduling system. The System Information Ratio $R^{t}$ is defined as follows:

(17) $$\begin{array}{c} R^{t}=\frac{I_{sch}^{t}}{I_{sys}^{t}}. \end{array}$$

According to Equations (13)–(16), expand and simplify Equation (17).

(18) $$\begin{array}{c} R^{t}=\sum_{b=1}^{x}\frac{rt\left(O_{n}^{kb}\right)}{dt\left(O_{n}^{kb}\right)} \end{array}$$

$∵U\left(O_{n}^{k}\right)\leq 1$, $∴R^{t}\leq x$. In order to describe the state of the manufacturing system based on information theory more conveniently, the standard information rate is defined as follows:

(19) $$\begin{array}{c} R_{nor}^{t}=\frac{R^{t}}{x} \end{array}$$

Equation (19) shows that the essence of $R_{nor}^{t}$ is the ratio of service capability of Production- Logistics Resources to the tasks of the manufacturing system. When $R_{nor}^{t}\leq 1$, the manufacturing system can ensure that all jobs are completed before the deadline. When $R_{nor}^{t}>1$, it is difficult for the manufacturing system to ensure that the completion time of all jobs is ahead of the deadline. From the perspective of information theory, it can be concluded that $R_{nor}^{t}$ can be used as the judgment basis of the current task requirements and serviceability of the manufacturing system. For processing speed and power consumption of manufacturing resources, the industry and academia have common sense. When manufacturing resources complete the same number of tasks, the operation speed is inversely proportional to energy consumption. This means that when tasks are assigned to a low-speed service group, power consumption will decrease while its processing time will increase, and vice versa. Considering that $R_{nor}^{t}$ is derived from the standard information entropy $E\left(O_{n}^{k}\right)$, the standard information entropy has the same mathematical meaning in theory. Therefore, this paper uses standard information entropy as the weight factor to balance energy consumption and time, such as Equation (12). The system can adjust the weight factor adaptively according to the task requirements and system status.

#### 4.3.4. Real-Time Production-Logistics Resources Adaptive Collaboration Strategy

**Figure 3:**
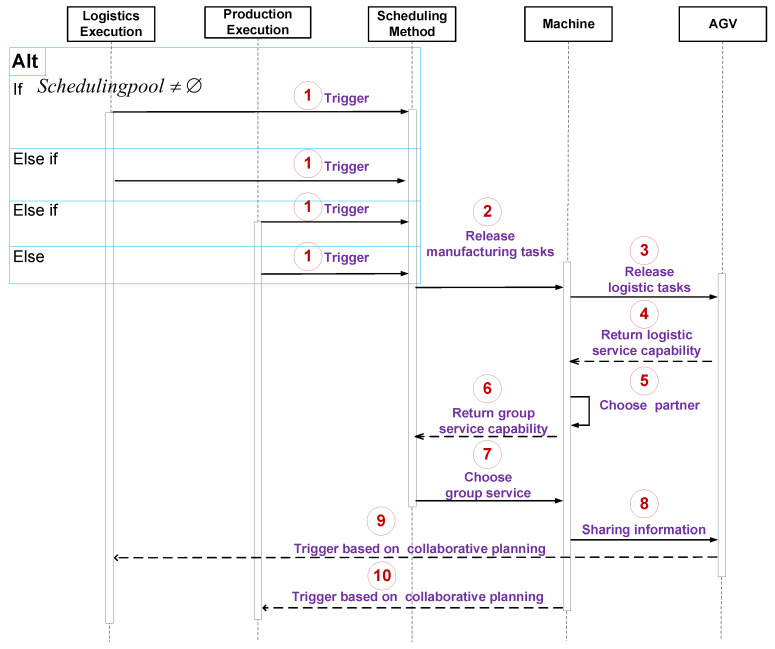
The Unified Modeling Language sequence diagram for the dynamic collaboration of Production-Logistics Resources.

When the production-logistics system oriented to real-time tasks is linked to the product, the execution of each production task will trigger the status change of the waiting tasks (as shown in Figure 3). At this point, if the scheduling pool is not empty, i.e., Schedulingpool≠⌀, the scheduling processes are listed below.

Step 1. The production task $O_{n}^{k}$ riggers the real-time reaction scheduling algorithm based on the trigger mechanism, and the scheduling algorithm is initialized. Schedule the task $O_{n}^{k+1}$ and obtain optional AGV resources set $\bar{V}$ and optional machine resources $\bar{M}$, according to the real-time information of production resources and logistics resources;

Step 2. The scheduling method sends the processing task to machines of all optional machines according to $\bar{M}$;

Step 3. Machines release logistics tasks to AGVs according to $\bar{V}$ and calculate their own service capabilities and calculate their service capabilities according to Equation (1);

Step 4. AGVs calculate their real-time service capabilities according to Equation (2) and return the results to the machines;

Step 5. The machines receive the real-time service capabilities of AGVs and calculate the group service capabilities according to Equations (7) and (8). Then each machine chooses the best partner (one AGV) as a group;

Step 6. Machines send the groups with real-time service capabilities to the scheduling method;

Step 7. The scheduling method selects the best service group as a service provider according to Equation (12), and return the scheduling plan to the service group leader (machine);

Step 8. The group leader sends the logistics scheduling plan to the group member (AGV);

Step 9. Logistics resources (e.g., AGVs) perform tasks according to the logistics scheduling plan;

Step 10. Production resources (e.g., machines) execute tasks according to the production scheduling plan. The algorithm at this time is over, and it waits to be triggered again and repeat steps (1) to (10).

## 5. Case Study

To verify the feasibility of the proposed approach in this paper, the case of a medium robot manufacturing company is studied. The performance of the proposed framework and the developed approach is evaluated. The interference factors in the actual production process are simulated and the effectiveness of the proposed approach is verified. Production-Logistics Resources with fuzzy attributes of operation time and customized orders with random arrival characteristics are considered.

All simulation examples were performed on a mobile computer with intel i7, 2.5 GHz CPU, and 8 Gb ram using python 3.7. The real-time data of WIPs and manufacturing resources in the smart job-shop were stored in the MySQL database. During real-time reaction scheduling, the python program communicates with the database through the Python DB-API interface.

### 5.1. Case Description

In the experimental scenario, the implementation process of the proposed method includes three parts. The main steps are as follows: (1) At the beginning of each simulation, jobs are randomly generated according to the job distribution and simulation duration. The job contains three groups of attributes: Start time, deadline, and production process. According to the start time of the job, the first task of the job is put into the scheduling pool, and the remaining tasks of the job are put into the task pool. (2) At this time, the scheduling pool is not empty, and the real-time reaction scheduling algorithm is triggered, as described in Section 4.3.4. (3) After that, repeat the process until all jobs are completed.

**Figure 4:**
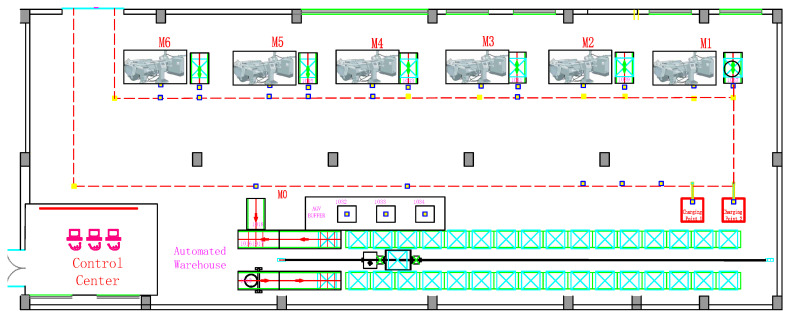
The simulation scenario.

As shown in Figure 4, the simulation scenario is a part job-shop of the robot manufacturing enterprise. There is a smart job-shop, 33 × 14 m, where there are six machines, some AGVs, and one warehouse.

The distance between the warehouse and machines are shown in Table 2. $m_{0}$ is warehouse, $m_{1}$ – $m_{6}$ are machines. In the beginning, the removable resources, such as AGVs, are in the same location of the warehouse.

In this case, each task has five processes. Table 3 gives the estimated processing time and power of each process by different machines.

In view of history data and current status of the factory, some necessary experimental settings are described below:

- The simulation period is 1800 s. That is, 15 jobs arrive at random within 30 min, (i.e., the arrival time of the jobs obeys Poisson distribution $p\left(\lambda \right)$, λ=0.5);
- The interval between the arrival time and the due date of jobs is 2000 s;
- In order to analyze the relationship between information utility and disturbance degree, three kinds of disturbance are set up in this paper: No disturbance $\left(z=0\right)$, slight disturbance $\left(z=0.1\right)$, and high disturbance $\left(z=0.2\right)$. In this form $tf_{1}=tf_{2}-z$ and $tf_{3}=tf_{2}+z$.

The idle power of machines is shown in Table 4. The transport power and speed of AGVs are shown in Table 5.

Based on the designed production scenario, the applicability of the proposed RSS approach is verified by the simulation experiment. Three performance indicators including makespan, energy consumption, and mean tardiness of work-piece, are considered in the study. In the scene of the designed experiment, each device and AGV can communicate, compute, and make decisions. The orders are released to the job-shop through the cloud manufacturing platform. They arrive at the manufacturing unit, and finally, adaptively are allocated to the production machines and handling equipment through the proposed approach. According to real-time production demand, production resources can calculate the capacity to provide service according to their current status and select the optimal Production-Logistics Resources combination to complete manufacturing tasks in real-time.

### 5.2. Results of Experiments

In this section, we first observe the relationship between information utilization and fuzzy disturbance in 12 different scenarios matched with random arrive jobs. Three typical production cases are found. Then, the typical production scenarios are simulated to observe the performance changes of three different indicators.

Comprehensively analyzing the relationship between interference degree and information utility in the intelligent job-shop is based on maximizing real-time information utility of intelligent job-shop. Three groups of numerical experiments with different disturbance degrees are simulated. The fuzzy operation time disturbance levels are divided into three types: No disturbance $\left(z=0\right)$, slight disturbance $\left(z=0.1\right)$, and high disturbance $\left(z=0.2\right)$. For each group of experiments, 15 samples were randomly generated according to the Poisson distribution. Then, the random samples are tested according to the four trigger strategies (i.e., APP, PW, ADP, and MSP) proposed in this paper. Therefore, three scenarios and four trigger strategies are considered in this paper. A total of 12 simulation results are shown in Table 6. In order to verify the marginal utility of AGVs, each result contains five sets of controlled trials, corresponding to 1 to 5 AGV, respectively. In order to test the accuracy, each scene is simulated 200 times. Table 6 shows the average of multiple simulation results.

As shown in Table 6, there are 12 subtables. In each subtable, there are five groups of experimental results, corresponding to different numbers of AGV. The unit of makespan $C_{max}$ and mean tardiness $\left(MT\right)$ is seconds, and the unit of total energy $\left(E\right)$ consumption is *J*. For each subgraph, it can be found that for one AGV, the energy consumption and makespan index are large, and the MT index is more significant than zero. When two AGVs are involved in the experiment, the *E* and $C_{max}$ index decrease rapidly, and the MT index becomes zero (except APP strategy with $\left(z=0.2\right)$ subgraph). When more than three AGVs are participating in the experiment, the decreasing range of each index slows down, which indicates that the marginal effect of AGV will be reached.

As shown in Table 6, the four subtables of each column have the same disturbance degree, and there are four trigger strategies from up to down: APP, PW, ADP, and MSP.In the first column, the subtables show the following results $\left(z=0\right)$: The APP strategy is better than other strategies, and marginal utility is achieved with three AGVs. The subtables in the second column $\left(z=0.1\right)$ show that ADP strategy is better than other strategies, and marginal utility is achieved with four AGVs. The subtables in the last column $\left(z=0.2\right)$ demonstrates that MSP strategy is better than other strategies, and marginal utility is achieved with five AGVs. Through the above analysis, we can draw the following conclusions: (1) With the increase of disturbance degree, the later trigger strategy is conducive to the optimization of scheduling. In other words, the utility of information is negatively correlated with the disturbance degree of the job-shop. (2) Increasing logistics resources can alleviate the disturbance to the production process to a certain extent. In other words, the number of logistics resources is positively correlated with the scheduling results, but it is not unlimited.

Compared with the Weight Method (WM) [24], Self-adaptive Collaboration Method (SCM) [25], and several traditional dynamic scheduling methods are used to demonstrate the real-time reaction scheduling performance of the proposed method in a highly disturbed production job shop. In this study, scheduling rules include the First In First Out (FIFO) dispatching rule, the Longest Processing Time (LPT) dispatching rule, and the Shortest Processing Time (SPT) dispatching rule. Based on the analysis of the relationship between interference and information utility, the comparative experiments are divided into three cases: (1) No disturbance $\left(z=0\right)$, APP strategy with three AGVs. (2) Slight disturbance $\left(z=0.1\right)$, ADP strategy with four AGVs. (3) High disturbance $\left(z=0.2\right)$, MSP strategy with five AGVs.

**Figure 5:**
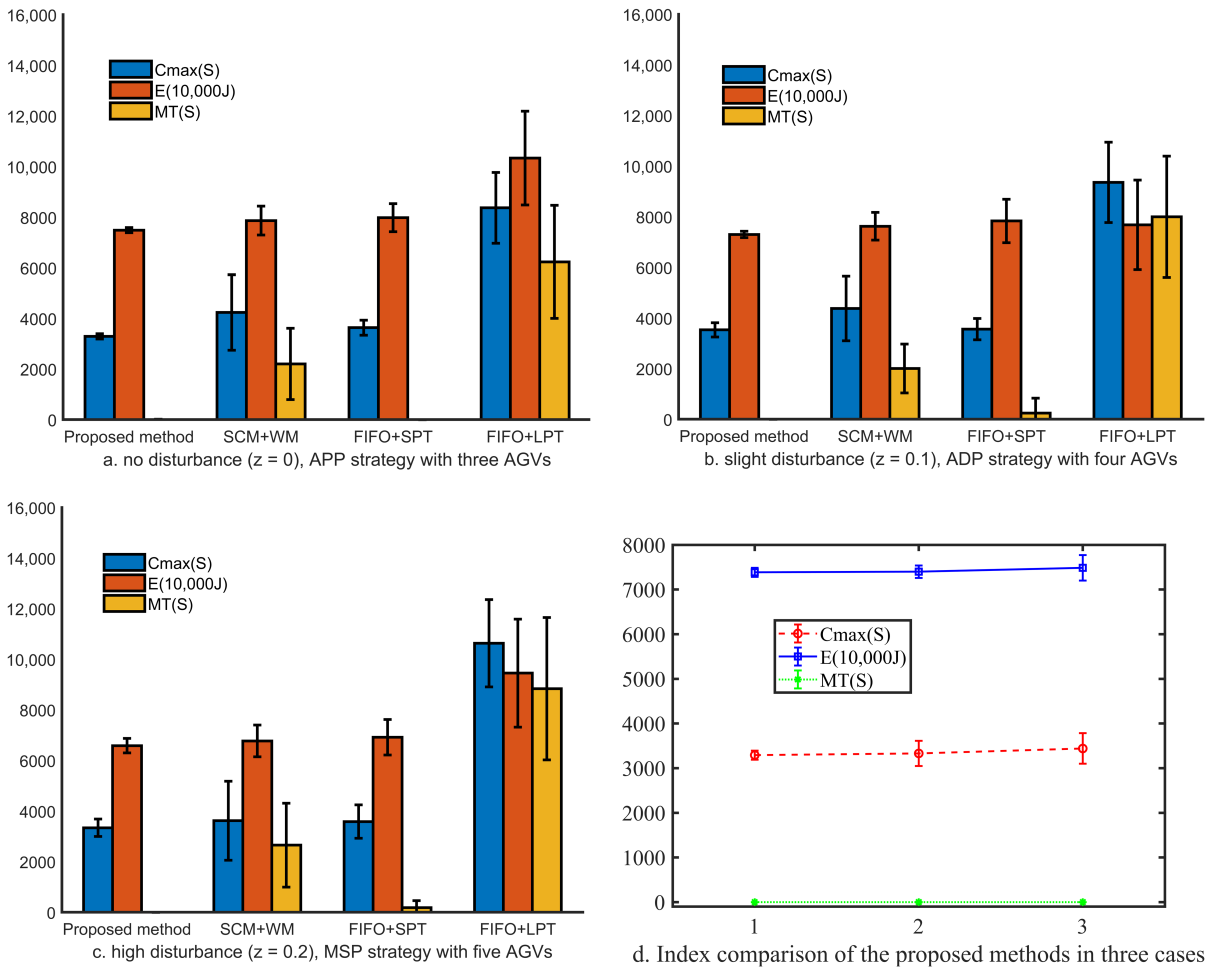
Experimental results in three cases.

For fairness of comparison, all comparison methods, as shown in Figure 5, are under the proposed algorithm architecture. That is to say, all the combination methods (i.e., SCM + WM, FIFO + SPT, and FIFO + LPT) are based on the dynamic collaborative strategy of Production-Logistics Resources. Figure 5 shows the mean and variance of 400 simulations of the proposed method and three combination methods in three cases. The three indicators, namely makespan, total energy consumption, and mean tardiness obtained by our proposed method are optimal. Figure 5a–c show that the mean and variance of indicators of the proposed method are minimal in all cases, which is consistent with the theoretical analysis. While the mean and variance of the three indexes are proportional to the fuzzy disturbance, the growth rates of the methods are different. Figure 5d shows that the performance of each index decreases as the disturbance increases. However, with the support of disturbance and information efficiency correlation theory, the extent of the decline is limited. In particular, the MT index of the proposed method is zero in all cases. These altogether demonstrate the efficiency and stability of the proposed method in dealing with a high disturbance production environment.

In order to adequately discuss the topic of the proposed approach’s transferability. We also simulated the assembly workshop of robot factory. The results show that the average and variance of the three indexes of the proposed method are minimum.

### 5.3. Discussion

As shown in Table 6, there is a potential correlation between the scheduling timing of new tasks and the degree of disturbance. With the increase of disturbance degree, the late triggered task has a better scheduling performance. There are two reasons for this phenomenon. On the one hand, due to the need to consider the production logistics collaboration, it is necessary to trigger tasks to find more suitable logistics resources to provide services. On the other hand, due to the fuzzy nature of working time, task allocation is not accurate. From this aspect, the later the task is allocated, the better the scheduling result will be. As shown in Figure 5, compared with other methods, the adaptive scheduling method proposed in this paper can better solve the random job arrival and fuzzy operation time in actual factories. Although the variance of energy consumption and makespan index increases with the increase of fuzzy degree, the average tardiness index of the proposed method can be kept at zero.

This case comes from a real robot job-shop. The purpose of this study is to solve some problems faced by the upgrading of the job-shop, such as the random arrival of customized orders and training of multi-skilled workers. Workers in the smart job-shop are cross-trained and implement most or all of the job shop [33]. The worker’s proficiency level will affect the working time when the man-machine cooperates, which is the origin of the fuzzy characteristic of the processing time. With the improvement of the proficiency of multi-skilled workers, the fuzzy degree of operation time will be lower. According to the research results of this paper, such as trigger strategy for maximizing information utility and adaptive scheduling method, it can reduce makespan, energy consumption and improve customer satisfaction. Case analysis shows that this approach has the potential to realize an intelligent and green society of a high dynamic job shop.

## 6. Conclusions and Future Work

In this article, a real-time adaptive scheduling method for production logistics resources was formulated to solve advanced scheduling problems with fuzzy processing time and random jobs arrival. Our method was used to solve the real workshop problem of production logistics collaborative scheduling under a high disturbance environment. One of the advantages of the proposed method is its versatility and scalability, although this paper only tested the scalability of logistics resources.

The scheduling problem considered in this paper has a high degree of complexity, which means that it is difficult to obtain the optimal solution quickly. This is the reason that we used the real-time task allocation method to schedule. To get the superiority and stability of global performance, we need to solve two problems: (1) When to allocate tasks and (2) how to allocate tasks.

In order to solve the above problems, this paper mainly carried out the following work:

Firstly, this paper analyzed the dynamic self-organization characteristics of Production-Logistics Resources in smart job-shop under the environment of the industrial Internet of Things and constructed a real-time capability model of Production-Logistics Resources. Then, it described the trigger mechanism based on the relationship between disturbance level and information utility. Finally, a scheduling approach oriented to real-time tasks was presented. An example verified the feasibility and effectiveness of the real-time reaction scheduling strategy. The results showed that this approach could effectively deal with the scheduling problem of real-time production tasks under high dynamic environment. This research has two main contributions. (1) The method of maximizing the information utilization rate of a dynamic job shop was found. (2) Real-time adaptive scheduling of high disturbance job-shop based on information entropy was realized.

In future work, the optimization of scheduling algorithm should be further studied based on dynamic resource allocation.

## Figures and Tables

**Table 1:** Notations used in the study.

Symbol	Definition
$job^{n}$	*n*-th job
$O_{n}^{k}$	*k*-th operation of $job^{n}$
$m_{j}$	The equipment number of machine *j*
$v_{i}$	The equipment number of AGV (Automated Guided Vehicle) *i*
$Am_{j}^{t}$	The capability model of $m_{j}$
$Av_{i}^{t}$	The capability model of $v_{i}$
$m_{j}^{s}$	The static properties of $m_{j}$
$m_{j}^{t}$	The real-time properties of $m_{j}$
$s_{j}^{t}$	Type of service that a machine can provide
$v_{i}^{s}$	The static properties of $v_{i}$
$v_{i}^{t}$	The real-time properties of $v_{i}$
$G_{U}^{t}$	The set of service groups at time *t*
$g_{y}$	The service group *y*, $g_{y}\in G_{U}^{t}$
$T^{y}$	The service time of $g_{y}$
$E^{y}$	The energy consumption of $g_{y}$
$\bar{M}$	Optional machine set
$\bar{V}$	Optional AGV set
$C^{n}$	Completion time of $job^{n}$
$d^{n}$	Due date for $job^{n}$
$a^{n}$	The arrival time of $job^{n}$
$L^{n}$	Tardiness of $job^{n}$

**Table 2:** The distances among the fixed resources.

Distance [m]	$m_{0}$	$m_{1}$	$m_{2}$	$m_{3}$	$m_{4}$	$m_{5}$	$m_{6}$
$m_{0}$	0	40	46	52	60	66	66
$m_{1}$	40	0	6	12	16	24	33
$m_{2}$	46	6	0	6	18	18	27
$m_{3}$	52	12	6	0	6	12	21
$m_{4}$	60	18	12	6	0	3	15
$m_{5}$	66	24	18	12	6	0	9
$m_{6}$	75	33	27	21	15	9	0

**Table 3:** Estimated processing time and power of each process.

Time [s]∖Power [KW]	$m_{1}$	$m_{2}$	$m_{3}$	$m_{4}$	$m_{5}$	$m_{6}$
job	180∖3.74	190∖3.11	170∖4.38	180∖4.24	190∖3.41	200∖4.5
	170∖4.38	190∖4.11	170∖4.48	170∖4.59	180∖4.24	200∖3.95
	360∖4.06	340∖3.18	370∖3.70	360∖4.08	350∖5.82	360∖4.08
	230∖4.18	240∖4.13	250∖3.20	230∖4.19	240∖4.09	200∖5.01
	220∖5.40	220∖5.39	240∖4.17	230∖5.28	240∖4.68	260∖4.57

**Table 4:** Idle power of machines.

$m_{j}$	$m_{1}$	$m_{2}$	$m_{3}$	$m_{4}$	$m_{5}$	$m_{6}$
Power [KW]	0.98	1.23	1.48	1.06	1.06	1.16

**Table 5:** Power and speed of the AGVs.

AGV	$v_{i}$
Power [KW]	1
Speed [m/s]	0.5

**Table 6:** Experimental results of four trigger strategies with three disturbance degrees.

**NA**	**APP Strategy with** ***z* = 0**			**APP Strategy with** ***z* = 0.1**			**APP Strategy with** ***z* = 0.2**		
	Cmax s	E J	MT s	Cmax s	E J	MT s	Cmax s	E J	MT s
1	3798	79,924,197	1299	3974	79,064,846	1497	5939	82,408,311	3968
2	3405	75,691,990	0	3510	74,218,272	0	3880	77,725,213	467
3	***3292***	***72,897,863***	***0***	3503	76,812,363	1	3713	76,131,173	193
4	3391	74,436,105	0	3667	75,481,459	8	3825	75,668,381	221
5	3365	74,412,437	0	3746	74,057,854	24	3837	77,256,997	314
**NA**	**PW Strategy with ** ***z*** ** = 0**			**PW Strategy with** ***z*** ** = 0.1**			**PW Strategy with ** ***z*** ** = 0.2**		
	Cmax s	E J	MT s	Cmax s	E J	MT s	Cmax s	E J	MT s
1	3822	79,330,556	1253	6089	84,452,674	2396	6218	84,428,615	1843
2	3792	74,608,693	123	3446	76,928,827	9	3893	78,549,737	22
3	3672	72,865,634	0	3327	75,986,267	0	3809	78,069,589	0
4	3575	73,633,627	17	3271	74,990,092	0	3837	77,075,180	24
5	3449	73,435,680	2	3244	74,585,985	0	3765	76,519,139	0
**NA**	**ADP Strategy with ** ***z*** ** = 0**			**ADP Strategy with ** ***z*** ** = 0.1**			**ADP Strategy with ** ***z*** ** = 0.2**		
	Cmax s	E J	MT s	Cmax s	E J	MT s	Cmax s	E J	MT s
1	5782	81,177,935	4808	3898	78,571,162	1624	3733	79,126,854	1479
2	3518	76,528,125	0	3535	76,166,753	0	3655	77,026,135	173
3	3334	68,141,735	59	3404	74,811,448	0	3766	78,408,417	354
4	3450	69,135,093	0	***3331***	***74,045,920***	***0***	3845	76,968,627	0
5	3399	67,960,194	44	3415	74,651,475	0	3886	76,126,530	0
**NA**	**MSP Strategy with ** ***z*** ** = 0**			**MSP Strategy with ** ***z*** ** = 0.1**			**MSP Strategy with ** ***z*** ** = 0.2**		
	Cmax s	E J	MT s	Cmax s	E J	MT s	Cmax s	E J	MT s
1	6147	84,598,977	3891	5987	84,610,784	2405	3998	78,673,637	2663
2	3749	76,817,096	517	3717	75,762,852	603	3575	77,627,778	104
3	3620	75,826,995	325	3551	76,028,675	219	3538	77,907,632	0
4	3658	68,436,910	135	3531	76,005,146	221	3476	76,699,788	0
5	3678	69,111,867	35	3529	75,609,530	198	***3441***	***74,860,718***	***0***

Notes: NA represents the number of AGVs; *E* represents total energy consumption; $C_{max}$ represents makespan; MT represents mean tardiness.
